# Diabetes Mellitus and Diabetic Kidney Disease: The Future Is Already Here

**DOI:** 10.3390/jcm12082914

**Published:** 2023-04-17

**Authors:** Alberto Martínez-Castelao

**Affiliations:** 1 Nephrology Department, Bellvitge University Hospital, Hospitalet, 08907 Barcelona, Spain; albertomcastelao@gmail.com; 2 GEENDIAB (Grupo Español de Estudio de la Nefropatía Diabética), Sociedad Española de Nefrología (S.E.N.), 39008 Santander, Spain; 3 RED in REN-RICORS 2040, Instituto Salud Carlos III, 28029 Madrid, Spain

Diabetes mellitus (DM) is a metabolic systemic disease with a high rate of morbidity and mortality. An editorial article in *The Lancet* in 2008 described the global challenge of diabetes as the biggest epidemic of the 21st century due to the increased percentage of type 2 DM (T2DM) and gestational DM cases and the number of young patients with T2DM (maturity-onset diabetes in young people, MODY) [1].

The disease burden of DM is expected to increase in the next few years and decades. The Atlas of the International DM Federation [2] estimated that 537 million adults (20–79 years) were living with DM in 2022, and this number could increase to 643 million by 2030 and 781 million by 2045. Almost seventy-five percent of adults with DM live in low- and middle-income countries. DM is estimated to have caused up to 6.7 million deaths in 2021 and led to costs of at least USA 966 billion, an increase of 316% in the last 15 years. Five million adults with impaired glucose tolerance are at increased risk for T2DM. More than 1 million children are living with type 1 DM [2].

In parallel with the IDF data, the number of publications related to DM on PubMed.gov is constantly increasing. As of the 3rd of April 2023, there were 419,974 relevant publications. There were 59,185 references to DM cardiovascular (CV) complications, 34,299 for diabetic nephropathy (DN) and 33,414 for diabetic kidney disease (DKD).

Age-standardized rates of DM-related complications, acute myocardial infarction, stroke and amputations have decreased in the last 15 years, but there has not been a corresponding reduction in the incidence of advanced chronic kidney disease (CKD) requiring renal replacement therapy (RRT). DKD continues to be the primary cause of end-stage renal disease (ESRD) worldwide.

The definition and the epidemiology of DN have evolved substantially. The classical presentation includes a progressive rise in albuminuria and subsequently proteinuria [3]. However, a non-proteinuric phenotype of patients with DM and CKD who develop deterioration of the estimated glomerular filtration rate (eGFR) is increasingly described [4]. In 2011, Tervaert et al. stated that the classical definition of DN includes glomerular lesions, but the wider concept of DKD considers renal involvement in DM patients, including tubulointerstitial and/or vascular lesions [5].

Novel instruments different from albuminuria or the eGFR, such as urinary proteomics, are necessary for better early detection of DKD in individuals with a baseline eGFR above 60 mL/min/1.73 m^2^.

Four decades ago, patients with DM attending nephrology outpatient clinics usually presented with advanced CKD, severe CV complications, advanced retinopathy or amaurosis or lower extremity amputations. However, DM patients frequently died due to uraemia or CV complications before reaching nephrology care.

**Multidisciplinary and multifactorial** care has dramatically changed the panorama this ancient, catastrophic disease. The pillars of an integrated approach for the management of patients with DM and CKD are based on five classes of drugs:

(1) The Angiotensin-Converting-Enzyme (ACE) inhibitors and Angiotensin II Receptor Blockers (AIIRBs), such as captopril, irbesartan or losartan. These were offered in some studies (captopril trial, IRMA II, IDNT and RENAAL) exploring the possibility of reducing albuminuria and the progression of renal damage. Thus, **RAAS inhibitors** are maintained as the first step of treatment for DM patients with hypertension and/or albuminuria or proteinuria.

(2) **Sodium-glucose-transporter 2** (**SGLT2**) inhibitors. Their discovery led to the introduction of these drugs to the usual hypoglycaemic therapy for adequate control in subjects with DM. They also offer a safe nephro and CV protection profile. The studies of Zinneman et al. and Wanner et al. with empagliflozin, the CANVAS programme with canagliflozin and many others have showed the possibility of achieving an optimal hypoglycaemic management while allowing CV and renal protection.

The meta-analysis by Toyama et al. [6] gathered data from 27 studies considering SGLT2 inhibitors. In 7363 patients with T2DM and CKD, SGLT2 inhibitors lowered glycated haemoglobin as well as blood pressure, body weight and albuminuria. A reduction in the risk of CV death, nonfatal myocardial infarction or nonfatal stroke and heart failure was also observed, without a clear effect on all-cause mortality. The study showed an attenuation of the annual decline in eGFR and a risk reduction in the composite renal outcome (HR, 0.71; 95% CI, 0.53–0.95). CV and renal outcomes were improved without evidence of additional safety concerns [6].

In a randomized clinical trial (RCT), Heerspink HL et al. [7] studied the effect of dapagliflozin on 4304 patients with CKD with or without DM with an eGFR of 25 to 75 mL/min/1.73 m^2^ and a urinary albumin-to-creatinine ratio (UACR) of 200 to 5000 mg/g. These patients received dapagliflozin (10 mg once daily) or a placebo. After a median of 2.4 years of follow-up, a primary outcome event of a composite of a sustained decline in the eGFR of at least 50%, end-stage kidney disease or death from renal or cardiovascular causes occurred in 197 (9.2%) of patients (n = 2152) in the dapagliflozin group and 312 (14.5%) of patients (n = 2152) in the placebo group (HR, 0.61; *p* < 0.00).The primary end-point was reduced in 44% (*p* < 0.001), and a composite of death from CV causes or hospitalization for heart failure was reduced in 29% (*p* = 0.009); 101 participants (4.7%) died in the dapagliflozin group and 146 participants (6.8%) died in the placebo group (HR, 0.69; *p* = 0.004). The effects of dapagliflozin were similar in patients with and without DM.

(3) **Glucagon-like peptide-1 receptor agonists** (**GLP-1RAs**). This is a class of incretin drugs that can improve CV and renal events in DKD. These hypoglycaemic agents can be safely used without an increased risk of hypoglycaemia in various CKD stages in patients with an eGFR above 15 mL/min/1.73 m^2^. These agents are also able to prevent the onset of albuminuria and retard the decline in GFR in diabetic patients, allowing weight reduction and CV benefits.

Some RCTs considering exenatide, liragluide, lixixenatide, dulaglutide, albiglutide and semaglutide showed positive results in the prevention of new-onset proteinuria, (ELIXA with lixisenatide or LEADER with liraglutide), persistent proteinuria (SUSTAIN-6 with semaglutide) or REWIND (with dulaglutide).

Semaglutide OW (1 mg vs. placebo) combined with RAAS inhibitors will be administered in an RCT to 3534 patients with DM meeting the following criteria: eGFR ≥ 50 to ≤75 mL/min/1.73 m^2^ and UACR > 300 to <5000 mg/g or eGFR ≥ 25 to <50 mL/min/1.73 m^2^ and UACR > 100 to <5000 mg/g. The primary objectives of the FLOW study are renal end-points, and this RCT is projected to be finished by the end of 2024 [8].

(4) **Antagonists of Endothelin-1** (**ET-1s**). Some, but not all studies on ET-1s have proved their ability to reduce albuminuria and proteinuria. Atrasentan has been shown to be able to reduce the risk of kidney failure but at the expense of increasing patients’ risk for edema and heart failure. Proteinuria can be reduced in patients with severe CKD, but the risk of heart failure may be increased. Waijer SW et al. [9] assessed the effects of atrasentan on kidney and heart failure events according to baseline eGFR and UACR in a post hoc analysis of the Study of Diabetic Nephropathy with Atrasentan (SONAR) trial. They studied the effect of atrasentan versus a placebo in 3668 patients with T2DM and CKD with elevated albuminuria. Atrasentan reduced the relative risk of the primary kidney outcomes—renal composite, heart failure, hospitalization (HR, 0.71)—consistently across all subgroups of baseline eGFR and UACR (*p* > 0.21). Patients in the highest UACR and lowest eGFR subgroups obtained the maximal benefit (*p* < 0.01). The risk of hospitalization due to heart failure was higher in the atrasentan group (HR 1.39). The relative and absolute risks of heart failure hospitalization were similar across baseline UACR and eGFR subgroups.

(5) The **Mineralocorticoid Receptor** (**MCR**) **antagonist, finerenone**. Very recently, finerenone was used in a RCT as an antifibrotic agent. The phase III studies FIDELIO-DKD and FIGARO-DKD [10] examined CV and kidney outcomes in patients with T2DM and CKD in different stages of CKD. Among 13,026 patients with a median follow-up of 3.0 years, the composite CV outcome occurred in 12.7% of the patients (n = 825) receiving finerenone and 14.4% (n = 939) of the patients in the placebo group (HR, 0.86; *p* = 0.0018). The composite kidney outcome occurred in 5.5% (n = 360) of patients receiving finerenone and 7.1% (n = 465) of those receiving the placebo (HR, 0.77; *p* = 0.0002). Hyperkalaemia as the cause of treatment discontinuation was more frequently observed in patients receiving finerenone (1.7%) than those given the placebo (0.6%) [10].

In a post hoc analysis of FIGARO-DKD, Ruilope LM et al. [11] observed that finerenone reduced the risk of CV events in patients with T2DM and stage 3 CKD. FIGARO-DKD included patients with a UACR of 30 to <300 mg/g and an eGFR of 25 to 90 mL/min/1.73 m^2^ or a UACR of 300 to 5000 mg/g and an eGFR ≥ 60 mL/min/1.73 m^2^. If we consider a decrease of >40% in the eGFR, a lower nonsignificant incidence rate was observed with finerenone compared with the placebo (HR = 0.87; *p* = 0.069). However, if we take into account a reduction in eGFR of >57%, a decrease in HR of 23% was obtained (*p* = 0.041).

A more intense effect of finerenone was observed in patients with severely increased albuminuria. Improvements in UACR, eGFR slope and CV risk were evident in both subgroups provided with finerenone.

The risk for kidney failure, atherosclerotic CV disease, heart failure and premature mortality is very high in patients with DM and CKD. As we previously established, recent clinical trials support new approaches to treating DM and CKD. The Kidney Disease: Improving Global Outcomes (KDIGO) 2022 and the 2022 American Diabetes Association (ADA) Standards of Medical Care in Diabetes and the Clinical Practice Guideline for Diabetes Management in Chronic Kidney Disease both provide evidence-based recommendations. A joint group of ADA and KDIGO representatives reviewed and developed consensus statements using both guidelines to guide clinical care.

On the basis of promoting a healthy lifestyle, consensus statements provide specific guidance on the use of RAAS inhibitors, metformin, SGLT2 inhibitors, GLP 1 RA and nonsteroidal MCR antagonists. The consensus provides clear recommendations for improving the clinical outcomes of people with diabetes and CKD [12].

If we take into consideration all these achievements, in my opinion, we are situated in a new era with regard to the integrated management of patients with DM and CKD. The future is not coming; the future is already here. The standards for management and clinical practice guidelines are precise. We can combine many new molecules for an adequate management of patients with DM and CKD. All these measures require a multidisciplinary approach that includes different specialists, patients and health providers in multidisciplinary teams and the establishment of educational programmes. These combined measures may aid the early diagnosis of both DM and CKD. Early referral of patients with DM and renal involvement to nephrology care is necessary to coordinate integrated multifactorial management and halt its progression to ESRD and CV damage [13].

## Data Availability

Not applicable.

## References

[1] Lancet T. (2008). The global challenge of diabetes. Lancet.

[2] International Diabetes Federation IDF Diabetes Atlas 2022 Reports. http://www.idf.org.

[3] Martínez-Castelao A., Navarro-González J.F., Górriz J.L., De Alvaro F. (2015). The concept and the epidemiology of diabetic kidney disase have changed in recent years. J. Clin. Med..

[4] Porrini E., Ruggenenti P., Mogensen C.E., Barlovic D.P., Praga M., Cruzado J.M., Hojs R., Abbate M., de Vries A.P., ERA-EDTA Diabesity Working Group (2015). Non-proteinuric pathways in loss of renal function in patients with type 2 diabetes. Lancet Diabetes Endocrinol..

[5] Tervaert T.W.C., Mooyaart A.L., Amann K., Cohen A.H., HCook H.T., Drachenberg C.B., Ferrario F., Fogo A.B., Haas M., de Heer E. (2010). Pathologic classification of diabetic nephropathy. J. Am. Soc. Nephrol..

[6] Toyama T., Neuen B.L., Jun M., Ohkuma T., Neal B., Jardine M.J., Heerspink H.L., Wong M.G., Ninomiya T., Wada T. (2019). Effect of SGLT2 inhibitors on cardiovascular, renal and safety outcomes in patients with type 2 diabetes mellitus and chronic kidney disease: A systematic review and meta-analysis. Diabetes Obes. Metab..

[7] Heerspink H.J.L., Stefánsson B.V., Correa-Rotter R., Chertow G.M., Greene T., Hou F.-F., Mann J.F.E., McMurray J.J.V., Lindberg M., Rossing P. (2020). Dapagliflozin in patients with chronic kidney disease. N. Engl. J. Med..

[8] Rossing P., Baeres F.M.M., Bakris G., Bosch-Traberg H., Gislum M., Gough S.C.L., Idorn T., Lawson J., Mahaffey K.W., Mann J.F.E. (2023). The rationale, design and baseline data of FLOW, a kidney outcomes trial with once-weekly semaglutide in people with type 2 diabetes and chronic kidney disease. Nephrol. Dial. Transplant..

[9] Waijer S.W., Vart P., Cherney D.Z.I., Chertow G.M., Jongs N., Langkilde A.M., Mann J.F.E., Mosenzon O., McMurray J.J.V., Rossing P. (2022). Effect of dapagliflozin on kidney and cardiovascular outcomes by baseline KDIGO risk categories: A post hoc analysis of the DAPA-CKD trial. Diabetologia.

[10] Agarwal R., Filippatos G., Pitt B., Anker S.D., Rossing P., Joseph A., Kolkhof P., Nowack C., Gebel M., Ruilope L.M. (2022). Cardiovascular and kidney outcomes with finerenone in patients with type 2 diabetes and chronic kidney disease: The FIDELITY pooled analysis. Eur. Heart J..

[11] Ruilope L.M., Pitt B., Anker S.D., Rossing P., Kovesdy C.P., Pecoits-Filho R., Pergola P., Joseph A., Lage A., Mentenich N. (2023). Kidney outcomes with finerenone: An analysis from the FIGARO-DKD study. Nephrol. Dial. Transplant..

[12] de Boer I.H., Khunti K., Sadusky T., Tuttle K.R., Neumiller J.J., Rhee C.M., Rosas S.E., Rossing P., Bakris G. (2022). Diabetes Management in Chronic Kidney Disease: A Consensus Report by the American Diabetes Association (ADA) and Kidney Disease: Improving Global Outcomes (KDIGO). Diabetes Care.

[13] Martínez-Castelao A., Soler M.J., Górriz Teruel J.L., Navarro-González J.F., Fernandez-Fernandez B., de Alvaro Moreno F., Ortiz A. (2020). Optimizing the timing of nephrologhy referral for patients with diabetic kidney disease. Clin. Kidney J..

